# Automated Three-Dimensional Microbial Sensing and Recognition Using Digital Holography and Statistical Sampling

**DOI:** 10.3390/s100908437

**Published:** 2010-09-09

**Authors:** Inkyu Moon, Faliu Yi, Bahram Javidi

**Affiliations:** 1 School of Computer Engineering, Chosun University, 375 Seosuk-dong, Dong-gu, Gwangju 501-759 South Korea; E-Mail: yifaliu@chosun.ac.kr; 2 Department of Electrical and Computer Engineering, U-2157, University of Connecticut, Storrs, CT 06269-2157, USA; E-Mail: bahram@engr.uconn.edu

**Keywords:** digital holography, 3D microscopy, cell analysis, statistical pattern recognition, medical imaging, bio-sensing

## Abstract

We overview an approach to providing automated three-dimensional (3D) sensing and recognition of biological micro/nanoorganisms integrating Gabor digital holographic microscopy and statistical sampling methods. For 3D data acquisition of biological specimens, a coherent beam propagates through the specimen and its transversely and longitudinally magnified diffraction pattern observed by the microscope objective is optically recorded with an image sensor array interfaced with a computer. 3D visualization of the biological specimen from the magnified diffraction pattern is accomplished by using the computational Fresnel propagation algorithm. For 3D recognition of the biological specimen, a watershed image segmentation algorithm is applied to automatically remove the unnecessary background parts in the reconstructed holographic image. Statistical estimation and inference algorithms are developed to the automatically segmented holographic image. Overviews of preliminary experimental results illustrate how the holographic image reconstructed from the Gabor digital hologram of biological specimen contains important information for microbial recognition.

## 1. Introduction

Optical imaging systems using digital holography under coherent illumination have been studied in three-dimensional (3D) display, medical diagnosis, 3D microscopy, robotics, defense, and security [1–19]. Digital holography [7] is attractive technique for the acquisition of 3D information for these varied applications.

Recently, information photonics-based optical sensing/imaging systems have been investigated for continuous, automated detection and identification of biological specimens [20–23]. The development of reliable, rapid and low-cost methods for sensing and identification of biological specimens is imperative. Such systems can be applied to medical diagnostics, environmental monitoring, food safety, early detection of biological weapons in security and defense.

Most conventional methods used to inspect and identify biological specimens typically involve time-consuming and labor-intensive biochemical assays or imaging and digital processing. Many imaging methods identify microorganisms based on specific two-dimensional (2D) shape information, image intensity color profile, and/or aggregation size and reaction time. However, a number of specimens such as protozoan cell structures, bacteria, and sperm tails are essentially fully transparent unless stained. This staining process is invasive for biological cells so that their viability can be adversely affected, which can be undesirable for certain studies of biological specimens. In addition, imaging methods often fail to recognize very minute differences in thickness, size, and shape. To overcome these obstacles, interferometry-based bio-sensing/imaging techniques have been developed to study biological specimens. In these techniques, the phase of a passing coherent light beam is changed by the differing densities and compositions within a biological specimen. Phase information can be recorded interferometrically, allowing for the study of biological specimens that would otherwise die from staining or be invisible to conventional imaging means. Here, optical sensing/imaging system integrated with information photonics for rapid, reliable sensing and identification of biological specimens is reviewed. This paper is an overview of the work we have done in real time identification of micro/nanoorganisms using 3D computational holographic imaging [20–23].

The Gabor digital holographic microscopy [24] described in this paper may be best method for obtaining the diffraction patterns of biological specimens with dynamic events, since it only requires a single exposure. It also automatically produces focused holographic images from the Gabor digital holograms of biological specimens without any mechanical scanning, as needed in conventional microscopy.

For the phase information acquisition of biological specimens, the magnified diffraction patterns of biological specimens are optically recorded by the presented Gabor digital holographic microscopy interfaced with a computer. Next, the magnified 3D image or stack of 2D images of a biological specimen is numerically reconstructed from the Gabor digital hologram by using the Huygens-Fresnel principle integral [25]. Since Gabor digital holographic microscopy provides the numerical reconstruction of many wave-fronts or sectional images of a biological specimen along the propagation direction using a single digital hologram, it is possible to obtain the information about how a biological specimen grows and migrates in the 3D space. Moreover, the better classification of biological specimens may be provided because both magnitude and phase information of them are reconstructed by the Gabor digital holographic microscopy.

For the automatic identification of biological specimens, the segmented areas of the reconstructed holographic images are used for selection of random test pixels used to build up the test statistic for recognition. It is more efficient to first filter out the unnecessary background from computationally reconstructed holographic images before feeding them into recognition modules. The segmentation helps finding regions of interest before processing for recognition. In this overview paper, the watershed image segmentation algorithm [26] is applied to the holographic images. Then, parametric or nonparametric inference algorithms are applied with the sample segment features randomly extracted from the segmented holographic image of the biological specimens.

These statistical sampling techniques allow for fast microbial identification and are found to be much more suitable in identifying the minute and morphologically simple species that are similar in their thickness, size and/or shape. Also, statistical hypothesis testing with the statistical sampling datasets can be applied to distinguish between different classes of biological microorganisms. These samples are processed using statistical inference algorithms for the equality of dispersions between the sampling segments of the reference and unknown input class holographic images. Statistical parametric and nonparametric estimators [27,28] can be used to analyze the difference of the ratio of variances of two populations, respectively.

The interferometery-based microscope discussed in this overview paper enables thickness measurements to be made that are not subject to these particular limitations, because with this technique the phase-change in the wavefront modulated by the specimen can be measured very accurately. Therefore the phase information for the specimens, which depends on the refractive index distribution of cellular cytoplasmic content and thickness of the specimen, can be measured in digital holographic microscopy. We believe, as our experiments show repeatedly, that biological organisms have their own unique characteristic phase distributions that can be exploited for their automatic identification.

## 2. Gabor Digital Holographic Microscopy

**Figure 1.**
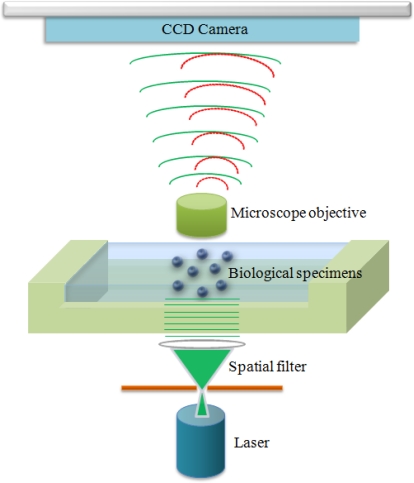
Experimental setup for recording the Gabor digital hologram of bio-specimens.

Gabor digital holographic microscopy [24] is described in the following section. The Gabor digital hologram or interference pattern of a biological specimen is recorded by a CCD (Charge-Coupled Device) array, as shown in Figure 1. Coherent light from an argon laser (center wavelength of 514.5 nm) is used as a source of illumination. A spatial filter and a collimating lens provide the spatial coherence. The planar coherent wavefront illuminates the biological specimen. Since biological specimens are semitransparent, the ballistic photons pass through the biological specimen without any scattering, which provides a reference beam for interferometry. The microscope objective captures and magnifies the reference beam and the transmitted diffracted wavefront on the hologram plane. The image sensor array at the location of the hologram plane captures the interference of the reference wave and the diffracted wavefronts from the biological specimen. The resulting interference patterns contain both the magnitude and phase information of the biological specimen.

After recording the Gabor digital hologram, a number of methods can be used for computational reconstruction of original bio-specimens including convolution and angular spectrum approaches [29]. In this overview paper, the angular spectrum method is applied to the Gabor digital hologram for 3D reconstruction of bio-specimens.

Let the field distribution of a biological specimen *O*(*x′, y′;z)* at the hologram plane or the Fresnel diffraction domain be given as [16]:

(1) $$O_{h}(x,y)=A_{h}(x,y)e^{j\phi_{h}(x,y)}=\int \int \int_{d-\delta /2}^{d+\delta /2}\left\{\frac{e^{j2\pi z/\lambda }}{j\lambda z}e^{j\frac{\pi }{\lambda z}(x^{2}+y^{2})}O(x^{\prime },y^{\prime };z)e^{j\frac{\pi }{\lambda z}({x^{\prime }}^{2}+{y^{\prime }}^{2})}e^{-j\frac{2\pi }{\lambda z}(xx^{\prime }+yy^{\prime })}\mathrm{dz}\mathrm{dx}\prime \mathrm{dy}\prime \right\}$$

Equation (1) represents the Fresnel transformation over a distance *d* with optical path difference *δ* along z-axis. The reference wave at the hologram plane is given as:

(2) $$R(x,y)=A_{r}\;(x,y)\;\text{exp}[j\phi_{r}(x,y)]$$

The interference pattern or Gabor digital hologram recorded at the CCD plane or hologram plane is represented as follows:

(3) $$I(x,y)={\left|O_{h}(x,h)+R(x,y)\right|}^{2}=A_{h}{\left(x,y\right)}^{2}+A_{r}^{2}+2A_{h}\;(x,y)A_{r}\;\text{cos}[\phi_{h}(x,y)-\phi_{r}]$$

where first term can be dropped because |*A_h_*(*x,y*)| << *A_r_* and the second term can be assumed as a constant. With the conjugate component of the Gabor digital hologram, ref. [30] demonstrated that crosstalk between real and conjugate terms are bound to low spatial frequencies in Fresnel Gabor digital holographic microscopy. Also ref. [23] showed that the conjugate component in the Gabor digital holographic microscopy can be neglected if many more fringe patterns of the biological specimen are captured by the CCD detector. Because of this condition, it can be assumed that the original focused image from the Gabor digital hologram is strongly dominant, whereas the defocused twin image overlapping the focused image is much weaker. Therefore, the field distribution of the original biological specimen from the Gabor digital hologram pattern can be calculated numerically by the following inverse Fresenl transformation or angular spectrum method with two Fourier transforms, which cancels the scale factor between the input and output:

(4) $$O(x^{\prime },y^{\prime })=\mathrm{IFrT}\{I(x,y)\}=\mathrm{IFT}\left(\mathrm{FT}\{I(x,y)\}\times \text{exp}\{j\pi \lambda d_{0}[\frac{u^{2}}{{\left(\Delta xN_{x}\right)}^{2}}+\frac{v^{2}}{{\left(\Delta yN_{y}\right)}^{2}}]\}\right)$$

where *d_0_* is reconstruction distance, *u* and *v* denote transverse discrete spatial frequencies, (Δ*x*, Δ*y*) is resolution at the hologram plane, and *N_x_* and *N_y_* are the whole hologram size in the *x*, *y* direction, respectively. Therefore, many wavefronts at arbitrary depth along the z-axis, including the one representing the biological specimen in focus, are computed from a single Gabor digital hologram. As we mentioned above, the reconstructed image from the Gabor digital hologram originally contains a conjugate image which degrades the quality of the reconstructed image. However, the some conjugate component in the background part of the reconstructed holographic image can be removed by using image segmentation algorithms. In addition the intrinsic defocused conjugate image also contains 3D information of the biological specimen for microbial identification purpose. As an additional merit, Gabor digital holographic microscopy allows one to obtain a dynamic time-varying scene digitally restored on the computer for monitoring and recognizing moving and growing micro/nano biological organisms. Our digital holographic microscopic system requires only a single exposure recorded for obtaining the diffracted pattern of a biological specimen. Therefore, the Gabor digital holographic microscopy can be suitable for recognizing moving biological cells and is robust to external noise factors such as fluctuation and vibration.

## 3. Statistical Sampling Method for 3D Identification of Biological Specimens

In the following, the design procedure to evaluate the microbial identification performance of the 3D sensing system based on Gabor digital holographic microscopy is described. For the automatic identification of bio-specimens, the segmented areas of the reconstructed holographic images are used for selection of random test pixels used to build up the test statistic for recognition. It is more efficient to first filter out the unnecessary background from computationally reconstructed holographic images before feeding them into recognition modules. The segmentation helps finding regions of interest before processing for recognition. In this overview paper, the watershed image segmentation algorithm has been used to efficiently remove the background part of the reconstructed image on the computer. Then, we randomly extract *n* pixels *m* times in the segmented holographic image. Each trial sampling segment consists of *n* complex values. Finally, parametric or nonparametric statistical inference algorithms are developed to identify biological specimens.

These statistical sampling methods allow for fast microbial identification and are found to be much more suitable in identifying the minute and morphologically simple species that are similar in their thickness, size and/or shape. Also, statistical hypothesis testing with the statistical sampling datasets can be applied to distinguish between different classes of biological microorganisms.

**Figure 2.**
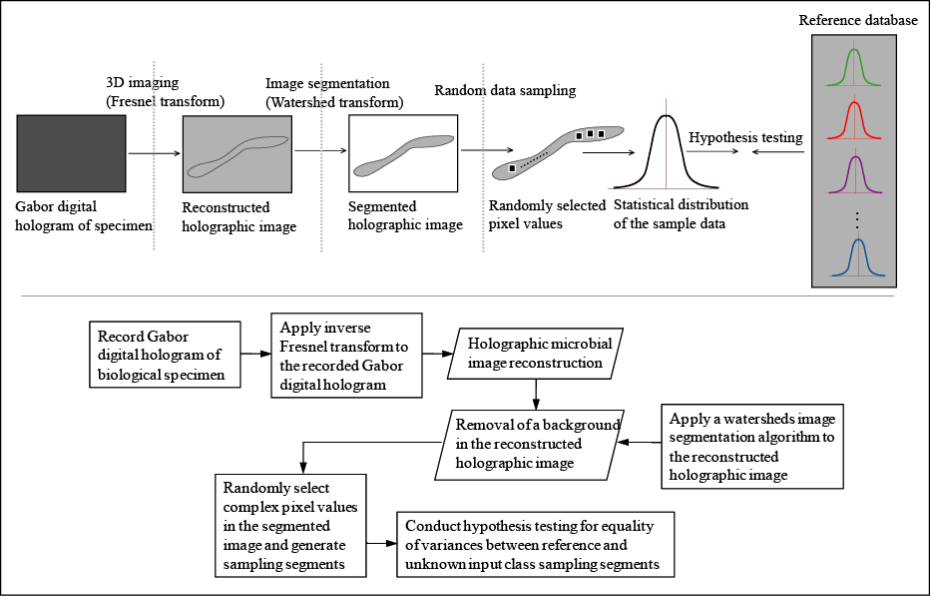
Statistical methodology to implement the presented three-dimensional microbial sensing/recognition system [22].

Our purpose of this overview paper is to illustrate that the digital holographic image or complex signal modulated by the specimen contains a rich data set for quantitative characterization and recognition of bio-specimens by using the statistical sampling methods and statistical hypothesis testing. Meanwhile, the advanced image recognition algorithms can be developed in order to improve the microbial identification performance. The statistical methodology for identification of biological specimens using digital holographic images is described in Figure 2 [22].

### 3.1. Parametric statistical inference algorithm

From the histogram analysis of the real and imaginary parts of the digital holographic image, it is assumed that the random variables (real or imaginary parts of the segmented holographic image) in the sampling segment nearly follow Gaussian distribution [22].

The statistical sampling distributions for the difference of parameters between the sample segment features of the reference and unknown input class digital holographic images can be calculated by using statistical estimation algorithms. The parametric statistical methods [27] are applied to the digital holographic images for a preliminary evaluation of the presented microbial identification system.

For comparing dispersion parameters, the sampling distribution of the ratio between two sample variances is computed. It is assumed that random variables *r* and *i* which are elements inside the reference and unknown input class sampling segments are statistically independent with identical Normal distribution $N(\mu_{r},\sigma_{r}^{2})$ and $N(\mu_{i},\sigma_{i}^{2})$. Also let *r* be independent of *i*. It is noted that the random variables *r* and *i* can be elements of the real part or the imaginary part of the reconstructed holographic image, so two separate univariate hypothesis testings [27] are performed.

For comparing the dispersion parameters between two sampling segments, we assume that all four statistical parameters are unknown and $\theta =(\mu_{r},\mu_{i},\sigma_{r}^{2},\sigma_{i}^{2})$. The ratio of the dispersions of two independent normal populations can be represented as follows [27]:

(5) $$F_{\left(n_{r}-1),(n_{i}-1\right)}=\frac{\{n_{r}/(n_{r}-1)\}V[r]/\sigma_{r}^{2}}{\{n_{i}/(n_{i}-1)\}V[i]/\sigma_{i}^{2}}$$

where *n_r_* and *n_i_* stand for sample size of the real part or the imaginary part of the reconstructed holographic image, respectively, *V*[·] is sample variance, and the *F* distribution has *n_r_* − 1 and *n_i_* − 1 degrees of freedom. Finally, a null hypothesis *H_0_* and an alterative hypothesis *H_1_* are defined as follows:

(6) $$H_{0}:\sigma_{r}^{2}=\sigma_{s}^{2},\;H_{1}:\sigma_{r}^{2}\neq \sigma_{s}^{2}$$

where the null hypothesis means that there is no difference between two population variances. Then for null hypothesis *H_0_*, Equation (5) can be given as [27]:

(7) $$F_{\left(n_{r}-1),(n_{s}-1\right)}=\frac{\left\{n_{r}/(n_{r}-1)\}V[r\right]}{\left\{n_{s}/(n_{s}-1)\}V[s\right]}=\frac{\hat{V}[r]}{\hat{V}[s]}$$

On the basis of a two-tailed test at a level of significance *α*, the following decision rules are defined [27]:

- Accept *H_0_* if the statistics, $\frac{\hat{V}[r]}{\hat{V}[s]}$ is placed inside the interval *F*_(*n_r_*−1), (*n_i_*−1),α/2_ to *F*_(*n_r_*−1), (*n_i_*−1), 1−α/2_.
- Reject *H_0_* otherwise.

The upper 100 × (*α*/2)% point of the *F*_(*n_r_*−1), (*n_s_*−1)_ distribution denotes *F*_(*n_r_*−1), (*n_s_*−1),α/2_. This decision rule implies that *H_0_* is true if the *F* distribution occurs between percentile value *F*_α/2_and *F*_1−*α/*2_ given the probability density function of the *F* distribution. *α* can be adjusted so that the probability of correct detection is 100 × (1−*α*/2)%. This is the area under the *F* distribution between *F*_α_2_/2_ and *F*_1−α_2_/2_. Thus the following probability can be claimed [27]:

(8) $$P\{F_{(n_{r}-1),(n_{s}-1),\alpha /2}^{-1}\times \left(\frac{\hat{V}[r]}{\hat{V}[s]}\right)<\frac{\sigma_{r}^{2}}{\sigma_{s}^{2}}<F_{(n_{r}-1),(n_{s}-1),1-\alpha /2}^{-1}\times \left(\frac{\hat{V}[r]}{\hat{V}[s]}\right)\}=1-\alpha$$

Finally, the statistical p-value is computed by empirical Monte Carlo techniques for the statistical decision to classify the specimen. It is a common practice to reject the null hypothesis if the calculated statistical p-value is less than 0.05. However, other cut-off p-values are also applicable, for example 0.01 or 0.10.

### 3.2. Nonparametric statistical inference algorithm

In the following, a statistical distribution-free test (KS-test) [28] is also employed for the comparison of two populations as nonparametric statistical test. In the previous section, it is assumed that the random variables (real or imaginary parts of the segmented complex holographic image) in the sampling segment nearly follow a Gaussian distribution. In this section, however, we use a nonparametric statistical test without any assumptions about the shapes of the underlying population distributions. The empirical cumulative density function (ECDF) can be obtained by the pixel values of the randomly selected *n* test pixel points. The test statistic for a null hypothesis is defined respectively by:

(9) $$\tilde{\Lambda }=E\left[{\left(f^{r}(u)-{\hat{f}}^{r}(u)\right)}^{2}\right]$$

where *f^r^* (*u*) and *f̂^r^* (*u*) are the ECDF obtained by the pixel values of the randomly selected *n* test pixel points from the reference holographic image. The possible values of |*f^r^* (*u*) − *f̂^r^* (*u*)| are in the range, 0 ≤ |*f^r^* (*u*) − *f̂^r^* (*u*)| ≤ 1. Similarly, the test statistic between a reference and unknown input class is defined by:

(10) $$\Lambda =E\left[{\left(f^{r}(u)-f^{i}(u)\right)}^{2}\right]$$

where *f^i^* (*u*) is the ECDF obtained by the pixel values of the randomly selected *n* test pixel points from the unknown input class holographic image. In order to obtain the statistical sampling distribution of the test statistics, the *f̂^r^* (*u*) and *f^i^* (*u*) are actually formed a number of times and then calculate the statistical distribution of the test statistics Λ̃ and Λ. It is noted that Λ̃ is the criterion discriminant function (CDF) appropriate for the null hypothesis. Finally, a statistical p-value is computed, *i.e.*, the probability that the variable with a probability density function for the null hypothesis is larger than the calculated statistic Λ for the statistical decision to identify biological specimens.

## 4. Experimental Results

In the following, the 3D visualization of micro/nano biological organisms using Gabor digital holographic microscopy is presented. In the experiments biological specimens were around several μm in size. Their Gabor digital holograms were recorded with a CCD array of 2,048 × 2,048 pixels and a pixel size of 9 μm × 9 μm, where the biological specimen was sandwiched between two transparent cover slips.

**Figure 3.**
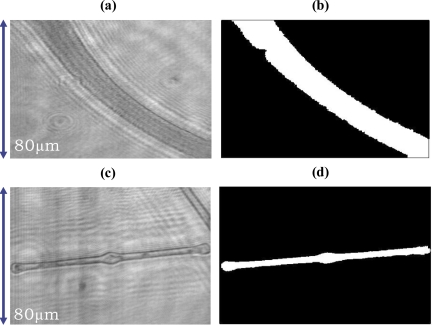
The microbial holographic images reconstructed at the distance 25μm from their Gabor digital holograms and binary windows for targets obtained by using a watershed image segmentation algorithm. **(a)** *Oscillatoria* bacteria. **(b)** Binary window for target (*Oscillatoria* bacteria). **(c)** *Diatom* alga. **(d)** Binary window for target (*Diatom* alga).

Figures 3(a,c) show *Oscillatoria* bacteria and *Diatom* alga holographic images reconstructed at the distances of 25 μm from their Gabor digital holograms, respectively, which were used to test the presented recognition system. For recognition purposes, a watershed image segmentation algorithm was used to remove the background parts in the reconstructed complex image. Figures 3(b,d) show the binary windows for targets (*Oscillatoria* bacteria and *Diatom* alga) obtained by using the watershed image segmentation algorithm.

**Figure 4.**
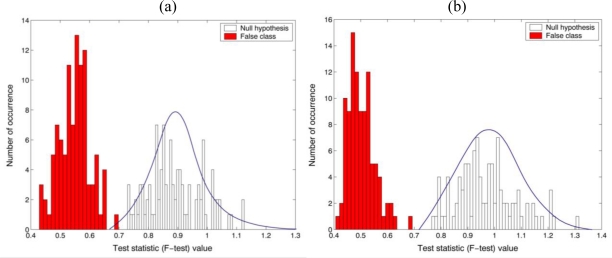
Parametric F-test results for the equality of two variances. 200 test pixel points were selected from segmented holographic image. **(a)** real part and **(b)** imaginary part in the reconstructed image. Reference: *Oscillatoria* bacteria. False class: *Diatom* alga.

Figures 4(a,b) shows the statistical distributions obtained from the parametric test statistic (F-test) for checking the equality of the variance between the reference (*Oscillatoria* bacteria) and the unknown input class (*Oscillatoria* bacteria or *Diatom* alga), where different specimen of same biological organism was tested for the true class. In order to measure the central tendency of the statistical sampling distribution of the parametric test statistic, 200 test pixel points were randomly selected from the segmented holographic dataset and then the trial sampling segments were generated 100 times for empirical Monte-Carlo experiments. Finally, two univariate F-test (real and imaginary parts in the reconstructed complex image) with the reference and unknown input class sampling segments have been separately conducted. As shown in Figures 4(a,b), it is noted that the parametric test statistic can discriminate between the two different datasets with 100% accuracy as the sample size was 200.

**Figure 5.**
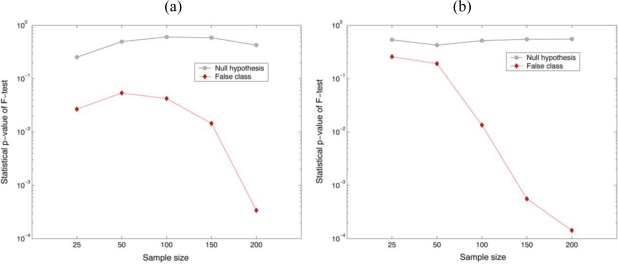
The average statistical p-value calculated from the parametric F-test. **(a)** real part and **(b)** imaginary part in the reconstructed image. Reference: *Oscillatoria* bacteria. False class: *Diatom* alga.

For preliminary evaluation of the recognition performance, a hypothesis testing [null hypothesis: $H_{0}(\sigma_{x}^{2}=\sigma_{y}^{2}=\sigma^{2})$] on the basis of a two-tailed test with a specific level of significance has been conducted with the parametric F-test values. It is shown in Figure 5 that the percentage of the correct matched sampling segments by the decision rule with 0.01 significance level for the null hypothesis (true class) was 100%, while for the false class was 0% when 200 random data samples selected from their own segmented holographic images. It is noted that the parametric test statistic can discriminate between the two different datasets with 100% accuracy as the sample size increase. These preliminary experimental results statistically illustrate that biological organisms have their own unique characteristic phase distributions that can be exploited for their automatic identification.

**Figure 6.**
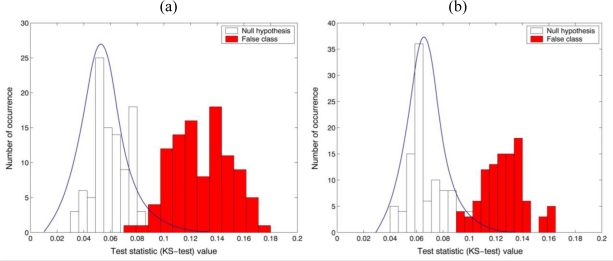
Nonparametric KS-test results for the equality of two variances. 200 test pixel points were selected from segmented holographic image. **(a)** real part and **(b)** imaginary part in the reconstructed image. Reference: *Oscillatoria* bacteria. False class: *Diatom* alga.

Figures 6(a,b) show the statistical distributions obtained from the nonparametric test statistic (KS-test) for checking the equality of the variance between the reference (*Oscillatoria* bacteria) and the unknown input class (*Oscillatoria* bacteria or *Diatom* alga). In order to measure the central tendency of the statistical sampling distribution of the test statistic, 200 test pixel points were randomly selected from the holographic dataset and then the trial sampling segments were 100 times generated for empirical Monte-Carlo experiments. Finally, two univariate KS-test (real and imaginary parts in the reconstructed holographic image) with the reference and unknown input class sampling segments have been separately conducted.

**Figure 7.**
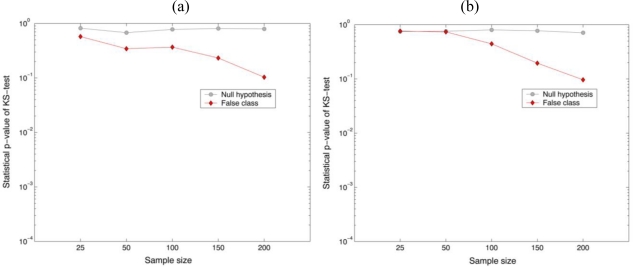
The average statistical p-value calculated from the nonparametric KS-test. **(a)** real part and **(b)** imaginary part in the reconstructed image. Reference: *Oscillatoria* bacteria. False class: *Diatom* alga.

For preliminary evaluation of the recognition performance, a hypothesis testing [null hypothesis: $H_{0}(\sigma_{x}^{2}=\sigma_{y}^{2}=\sigma^{2})$] has been conducted with the nonparametric KS-test values. It is shown in Figure 7 that the percentage of the correct matched sampling segments by the decision rule with 0.01 significance level for the null hypothesis was 100%, while for the false class was over 90% when 200 random data samples selected from their own segmented holographic images. As a result, it is noted that the parametric F-test can provide better performance to distinguish two different sampling segments than the KS-test.

**Figure 8.**
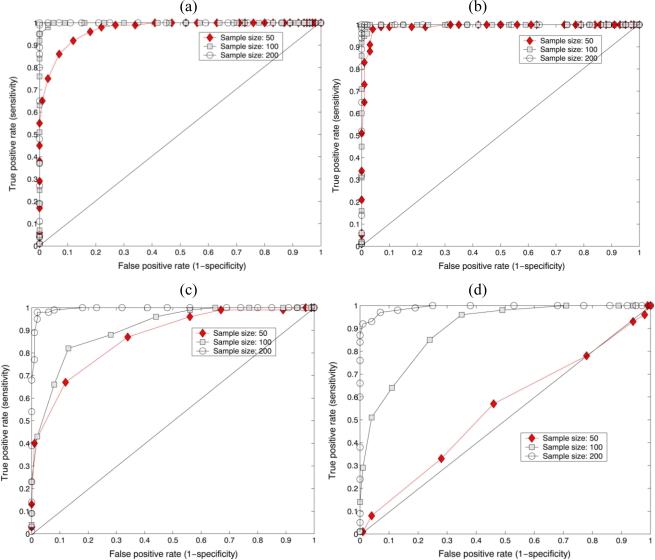
The ROC-curve results between reference and false class statistical distributions. The distributions are generated from the test statistic F-test and KS-test. Sampling segments are obtained from both real and imaginary parts in the reconstructed complex image. **(a)** ROC-curve result for real part in the complex image with F-test. **(b)** ROC-curve result for imaginary part in the complex image with F-test. **(c)** ROC-curve result for real part in the complex image with KS-test. **(d)** ROC-curve result for imaginary part in the complex image with KS-test. Reference: *Oscillatoria* bacteria. False class: *Diatom* alga.

Figures 8(a,b) show the ROC curve results between the reference (*Oscillatoria* bacteria) and false class (*Diatom* alga) statistical sampling distributions obtained from the parametric test statistic (F-test). The number of the test pixel points varies from 50 to 200. The well-focused holographic images for the reference and the false class were reconstructed from their Gabor digital holograms, respectively. The accuracy of the parametric test statistic depends on how well the test separates the two groups into those with similar properties or dissimilar ones. The closer the curve follows the left-hand border and then top border of ROC space, the more accurate the test. As shown in Figure 8, the area under the ROC curve approached 1, which signifies a perfect test as the sample size increased.

Figures 8(c,d) show the ROC curve results between the reference (*Oscillatoria* bacteria) and false class (*Diatom* alga) statistical sampling distributions generated by using the nonparametric KS-test statistic. The number of the test pixel points varies from 50 to 200. The well-focused holographic images for the reference and the false class were reconstructed from their Gabor digital holograms, respectively. As shown in Figures 8(c,d), the area under the ROC curve approached 1, which signifies a perfect test as the sample size increased. It is illustrated, as our experiments show repeatedly, that digital holographic image modulated by the specimen contains a rich data set for quantitative characterization and recognition of biological specimens with the statistical sampling methods. We have directly applied parametric or nonparametric statistical algorithms on the sample segment features of the segmented holographic image of the biological specimens for their identification. These statistical techniques are found to be much more suitable in identifying the minute and morphologically simple species that are similar in their thickness, size and/or shape. We believe that Gabor digital holography based automated microbial identification system which interweaves the complex amplitude wavefront modulated by the specimen with statistical methodology leads to fast and reliable differentiation of transparent biological specimens.

## 5. Conclusions

Automated micro/nano biological organism sensing and recognition system using Gabor digital holographic microscopy and a statistical inference has been overviewed. 3D sensing is based on Gabor digital holographic microscopy. In order to evaluate the recognition performance of the presented microbial sensing system, the Gabor digital holograms of biological specimens have been optically measured and then the complex holographic images of the original biological specimens have been digitally reconstructed with the recorded Gabor digital hologram. Target sampling segments have been extracted in the segmented holographic image after applying watershed image segmentation algorithm to the reconstructed holographic image. The sampling probability distribution of the difference of the ratio of the dispersions have been calculated between the reference and unknown input class sampling segments varying the sample size of sampling segment. Finally, the presented sensing system has been tested by performing hypothesis tests for the difference of the ratio of variances with a statistical decision rules. It has been shown in preliminary experiments that the holographic image reconstructed from only a single Gabor digital hologram of biological specimen contains important information for recognition and classification and they may be identified using a statistical estimation and inference algorithms. The shapes of some bacteria and algae are filamentous, spherical, and branched. They may look similar in terms of shape. This approach allows the presented system to be tolerant of shape in recognizing biological specimens like bacteria or algae.
